# Isolation, identification and characterization of *Lactobacillus* species diversity from *Meekiri*: traditional fermented buffalo milk gels in Sri Lanka

**DOI:** 10.1016/j.heliyon.2021.e08136

**Published:** 2021-10-06

**Authors:** A.M.M.U. Adikari, Hasitha Priyashantha, J.N.K. Disanayaka, D.V. Jayatileka, S.P. Kodithuwakku, J.A.M.S. Jayatilake, J.K. Vidanarachchi

**Affiliations:** a Department of Food Science and Technology, Wayamba University of Sri Lanka, Kuliyapitiya, Sri Lanka; b Department of Molecular Sciences, Swedish University of Agricultural Sciences, Box 7015, SE-750 07 Uppsala, Sweden; c Department of Animal Science, Faculty of Agriculture, University of Peradeniya, Peradeniya 20400, Sri Lanka; d Department of Agricultural Biology, Faculty of Agriculture, University of Peradeniya, Peradeniya 20400, Sri Lanka; e Department of Oral Medicine and Periodontology, Faculty of Dental Sciences, University of Peradeniya, Peradeniya 20400, Sri Lanka; f Department of Obstetrics and Gynecology, LKS Faculty of Medicine, The University of Hong Kong, Pok Fu Lam, Hong Kong

**Keywords:** *Meekiri*, Buffalo milk, Lactic acid bacteria, 16S rRNA sequencing, Probiotic

## Abstract

Traditional fermented buffalo milk gel; *Meekiri*, is a popular buffalo milk-derived product in Sri Lanka. Predominantly, it is produced using the back-slopping (adding a small amount of the previous fermentate) technique, following the life-long traditions available at the cottage level. Hence, diverse and unclassified starter cultures are likely to be established across the varying geographical regions of *Meekiri* production. In the present study, we aimed to elucidate the diversity of lactic acid bacteria (LAB) and their characteristics including probiotic properties from major *Meekeri* production areas (n = 22) in Sri Lanka. Lactic acid bacteria was isolated from locally produced *Meekiri* samples (n = 23) and characterized based on morphological, biochemical, physiological profiles and potential of probiotic properties. The isolates revealed five different colony and cell morphologies and were classified as heterofermenters, homofermenters and facultative heterofermenters based on CO_2_ production using glucose. None of the isolates showed the ability to grow either at 5 °C or 0 °C, while 71 % and 100 % survival of the isolates were observed at 15 °C and 45 °C, respectively. Amplified ribosomal DNA restriction analysis (ARDRA) primarily grouped the isolates into three distinct clusters based on their DNA banding patterns. Subsequently, 16S rRNA sequencing of isolates revealed the presence of four species namely, *Limosilactobacillus fermentum* (n = 18), *Latilactobacillus curvatus* (n = 2), *Lactobacillus acidophilus* (n = 2) and *Lactiplantibacillus plantarum* (n = 1) and in the phylogenetic analysis, it was represented by four distinctive clades. All the isolated species demonstrated promising probiotic potential with antibiotic sensitivity, antimicrobial properties, bile acid tolerance and acid tolerance. In conclusion, traditional back-slopping *Meekiri* in Sri Lanka contains diverse LAB, with a negligible geographical variation at species-level. Our work provides a strong foundation and insights into future applications in starter culture development for the fermentation of buffalo's milk.

## 1 Introduction

Fermented buffalo milk production is popular in countries where buffalo rearing is practiced. Among them, the Philippines, West Sumatra, Indonesia, India and Sri Lanka are very popular for their fermented buffalo milk products, such as *Kesong puti*, *Dadih*, *Dahi*, *Lassi*, *Shrikhand* and *Meekiri* (Anggraini et al., 2019; Abesinghe et al., 2020)*. Meekiri* is a traditional fermented milk gel derived from buffalo milk in Sri Lanka. Fresh buffalo milk is heat-treated (pasteurized at around 92 °C) and inoculated (inoculation rate is undefined in cottage-level production) with lactic acid bacteria (at 42 °C), and incubated overnight at room temperature (∼30–35 °C) to achieve milk coagulation (Abesinghe et al., 2020).

In Sri Lanka, considering the technique and scale of production, *Meekiri* production ranges from cottage to commercial level. At the cottage level, fermentation is achieved using a small portion of inoculum from the previous-day coagulum (i.e. back-slopping) (Abesinghe et al., 2020). The popularity of using back-slopping to produce fermented buffalo milk products in rural context is mainly due to its simplicity with respect to input (e.g. required only a small portion of previous day milk gel) and logistics (e.g. not maintaining specialized or dedicated storage facilities for starter cultures and usage of natural utensils such as bamboo and banana leaves (Harnentis et al., 2019). Given that the inoculum is selected from a previous-day product based on optimal product quality characteristics, back-slopping could essentially shorten the initial phase of the fermentation process and lower the incidences of product quality failure (Gastrow et al., 2020).

In Sri Lanka, small-scale *Meekiri* production is concentrated to the major buffalo farming areas and standardized commercial starter cultures are not used for the fermentation process. Therefore, a, diverse and specific microbial consortium might be present in *Meekiri* produced at varying geographical regions. Microbiota in different fermented dairy products consists of a wide variety of lactic acid bacteria (LAB), such as *Leuconostoc, Lactobacillus, Streptococcus*, *Lactococcus, Bifidobacterium* etc. (Gastrow et al., 2020; Khedid et al., 2009; Venema and Surono, 2019), and eumycetes (e.g. yeasts) (Maoloni et al., 2020). The genus *Lactobacillus* has been further classified into 23 novel genera including *Limosilactobacillus, Latilactobacillus,* and *Lactiplantibacillus* (Zheng et al., 2020)*.* Recently, various LAB species have gained increased recognition as probiotics. Thus, naturally occurring LAB species in *Meekiri* could serve as probiotics and become a key ingredient in the functional food market. *Lactobacillus* species are with most key probiotic properties such as tolerance to acid and bile, resistance to antibiotics and the antimicrobial activity (Shuhadha et al., 2017). A detailed study on the diversity of LAB in back-slopping *Meekiri* available in Sri Lanka has not been conducted previously and scarce information is available regarding their characteristics and properties*.* Therefore, the present study aimed to isolate and evaluate the diversity of LAB species sourced from major *Meekiri* producing regions of Sri Lanka to characterize their biochemical, physiological and probiotic properties. We hypothesized that the cottage-level *Meekiri* produced in Sri Lanka using back-slopping will carry diverse LAB species with varying properties and characteristics associated with the geographical variations of the production locations.

## 2 Materials and methodology

### 2.1 Experimental design and sampling procedure

**Figure 1:**
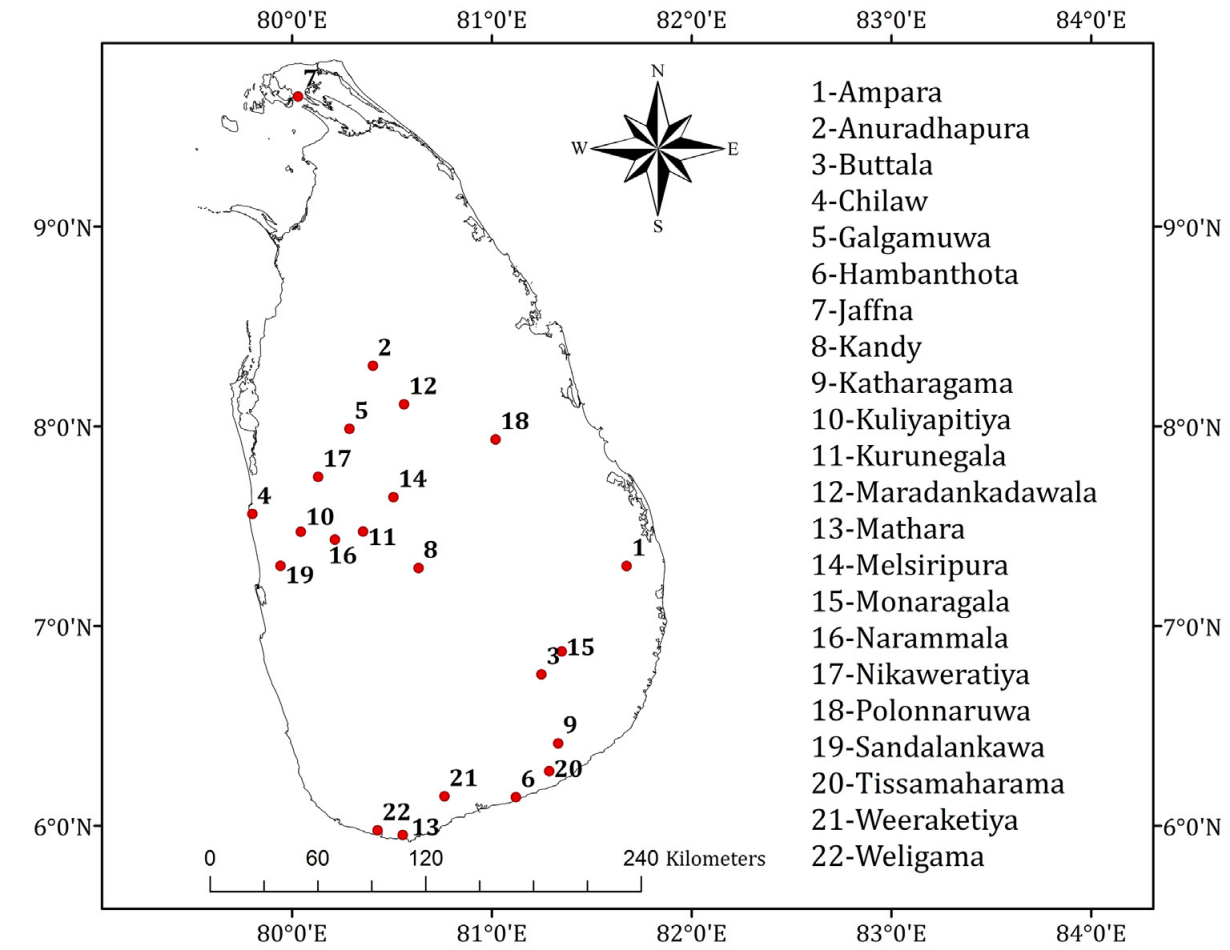
Sampling sites of Sri Lankan back-slopping *Meekiri.*

Fresh back-slopping fermented milk gel samples (n = 23) were collected from small-scale (e.g. household level) producers in 22 different locations covering the major *Meekiri* producing areas in Sri Lanka (Figure 1). As per practical reasons, two samples from each location were collected. Samples were transported to the laboratory at room temperature and analyzed for LAB in the same sequential order as stated below in duplicates.

### 2.2 Isolation and purification of bacteria from *Meekiri*

The isolation and purification of bacteria from *Meekiri* on de Man-Rogosa-Sharpe agar (MRS; Oxoid, Cambridge, UK) was performed according to the method described by Pathak and Dutta (2016) and Vanniyasingam et al. (2019) with modifications. From each sample, 5 g was homogenized with sterile 10 mL of distilled water. An inoculum from the homogenized sample was placed on MRS agar. Triplicate plates were inoculated following streak plate technique and incubated at 37 °C in an airtight plastic container for 72 h with 5–10 % CO_2_ using AnaeroGen (Oxoid Ltd. AN0025A). Morphologically distinctive, well-isolated colony from each sample were picked and further streaked into fresh MRS plates until a pure culture was obtained. Pure cultures were preserved by suspending the bacterial cells in MRS broth containing 10 % (v/v) sterilized glycerol and stored at -80 °C for further studies.

### 2.3 Biochemical and physiological characterization

Using fresh cultures of LAB (isolates grown on MRS agar for 24 h) biochemical and physiological characterizations were performed according to the methods described by Khedid et al. (2009) and Shabana et al. (2013) with modifications.

#### 2.3.1 Catalase test

The catalase test was conducted to identify microbes possessing catalase enzymes, which catalyze the decomposition of hydrogen peroxide. The formation of oxygen bubbles was evaluated in duplicates by mixing a loopful of bacteria with a drop of 3 % (v/v) hydrogen peroxide on a microscopic slide.

#### 2.3.2 Motility test

The motility test was performed using the hanging drop method to identify the isolates as motile or non-motile species. First, a small drop of MRS broth containing a particular isolate was placed in the middle of a cleaned cover glass. Afterwards, a concave depression slide was mounted to the cover glass using Vaseline where the cavity of the slide is facing downwards onto the broth drop. Subsequently, the slide was turned upside down and observed (×400) using a Primo Star iLED microscope (Carl Zeiss, GmbH) in duplicates.

#### 2.3.3 Endospore test

Production of spores was observed to identify and classify bacteria as spore formers or non-spore formers. A bacterial smear was prepared on a microscopic slide and coloured with malachite green and safranin for 2 min and 30 s. The excess stain was washed by rinsing with water in between the staining and the slide was observed (×1000) using Primo Star iLED microscope (Carl Zeiss, GmbH) under oil immersion.

#### 2.3.4 Voges-Proskauer test

This test determines whether the LAB produces acetoin, a precursor of 2,3-butanediol produced as a fermentation byproduct from glucose. One colony of each isolate was inoculated into 5 mL of freshly prepared sterile Methyl-red Voges-Proskauer broth and incubated for 48 h at 37 °C in duplicates. Barritt's A reagent (0.6 mL) and B reagent (0.2 mL) were added into the broth and the colour change was observed after 15 min. A positive reaction was indicated by the development of pink colour.

#### 2.3.5 Hydrogen sulfide production

Hydrogen sulfide production is a property of bacteria, containing sulfur-reducing compounds. Production of hydrogen sulfide was detected by stabbing one colony of each isolate into Triple Sugar Iron Agar (TSI) slants (HiMedia, M0211), which were prepared according to manufacturer's instructions and incubated at 37 °C for 24 h in duplicates, with a non-inoculated tube considered as the negative control. A positive reaction was indicated by the development of a black colour.

#### 2.3.6 Hugh and Leifson's oxidation fermentation test

Hugh and Leifson's oxidation fermentation test was conducted to determine whether an isolate metabolizes carbohydrates by oxidation, fermentation, or non-saccharolytic way (cannot use the carbohydrates in the medium). First, Hugh and Leifson's agar was poured into two 10 mL centrifuge tubes, allowed to solidify as a butt, and inoculated with one colony from each isolate. One of the tubes was covered with 1 mL sterile liquid paraffin to prevent diffusion of oxygen to create an anaerobic condition and the other tube was loosely covered, allowing oxygen to be in contact with the medium. Afterwards, both tubes were incubated at 37 °C for 72 h in duplicates. *E. coli* and *Pseudomonas aeruginosa* were used as positive controls to detect fermentation and oxidation reactions, respectively.

#### 2.3.7 Gas production from glucose fermentation

The ability to ferment glucose was determined to classify isolates as homofermenter, heterofermenter or facultative heterofermenter. One colony of each isolate was inoculated into centrifuge tubes containing modified MRS broth (prepared with 20 g of glucose in 1 L of broth) which contain inverted Durham tubes and incubated at 37 °C for 24 h in duplicates. A non-inoculated tube was kept as the negative control and *E. coli* was used as the positive control.

#### 2.3.8 Production of acids from selected carbohydrates

3The ability of the isolates to ferment eleven different sugars was tested given LAB use different carbohydrates as their substrates. Fresh bacterial cell pellets were harvested by centrifuging (10,000 *×g* for 10 min) MRS broth in which the isolates were grown for 24 h and the pellets were washed with sterilized distilled water followed by suspending in 5 mL of modified MRS broth (prepared using the ingredients present in commercially available MRS broth without glucose) with bromecresol purple. Eleven different sugar solutions (i.e. arabinose, galactose, lactose, maltose, mannitol, melezitose, melibiose, raffinose, salicin, sorbitol and trehalose) at a concentration of 10% (w/v), except for salicin (5% w/v) were prepared. Forty microliters of sugar solution and 160 μL of suspended cells were transferred (1% w/v final sugar concentration for salicin and 2% w/v final sugar concentration for others) to a 96-well microtiter plate and incubated for 24 h at 37 °C. The study was conducted in duplicates with the same conditions. Glucose and suspended cells were used as the positive control and only suspended cells of each isolate were used as the negative control.

#### 2.3.9 Temperature tolerance test

The ability of the isolates to grow in different temperatures was studied using 1% (v/v) of each isolate in 10 mL of MRS broth containing 0.4% bromocrysol purple. Isolates were incubated at different temperatures (e.g. 5, 10, 15, and 45 °C) for one week in duplicates.

#### 2.3.10 NaCl tolerance test

Ability to grow under NaCl concentrations was tested using 1 % (v/v) of isolates in 10 mL of MRS broth containing 0.4% bromocrysol purple adjusted with different NaCl concentrations (e.g. 2%, 4%, and 6.5 % w/w) and incubated at 37 °C for one week in duplicates.

### 2.4 Molecular identification of the isolates with amplified ribosomal DNA restriction analysis (ARDRA) of 16-23S rDNA

The PCR amplicons of 16-23S rDNA intergenic spacer regions of the isolates were digested with the restriction endonuclease *HaeIII* enzyme (Promega, USA) according to Vidanarachchi et al. (2007) and the banding patterns were identified through a gel documentation system (Vilbour Lourmart, France).

### 2.5 Sequencing of 16S rRNA gene of the isolates and phylogenetic analysis

Fresh bacterial cells were harvested by centrifugation (10,000 *×g* for 10 min) of 25 mL of MRS broth in which bacteria were grown for 24 h. Genomic DNA was extracted using Wizard® genomic DNA purification kit (Promega, USA) according to the manufacturer's guideline. The 16S rRNA gene region of the DNA was amplified using the primer pair *TH008* (forward 5′AGRGTTYGATTMTGGCTCAG 3′) and *PH1522* (reverse 5′ AAGGAGGTGATCCAGCCGCA 3′), with an initial denaturation cycle of 1-minute at 95 °C, followed by 30 cycles of 30 s denaturation at 95 °C, 30 s annealing at 57 °C and 45 s elongation at 72 °C and a final elongation of 10 min at 72 °C (Vidanarachchi et al., 2007).

The products were resolved on a 1% agarose gel and were visualized after post staining with ethidium bromide. Amplified DNA was purified and concentrated using Wizard® SV gel according to the manufacturer's instructions and PCR clean-up system (Promega, USA). Sequencing was performed using an ABI3730XL DNA sequencer (Applied Biosystems, USA) and the resulting gene sequences [GenBank accessions: MN960277 to MN960299] were used in a homology search in GeneBank (National Center for Biotechnology Information, USA) using the Basic Local Alignment Search Tool (BLAST) algorithm. The species assigning was done based on the best hit, that carried the highest sequence similarity, with 100% query coverage and a Eugan value closer to 0.

Based on Jalilsood et al. (2015), the 16S rRNA gene sequence of the sister species *Bacillus subtilis* [GenBank accession: NR112116] was retrieved from GenBank and was considered as the outgroup for the reconstruction of the phylogeny. Multiple sequence alignment of the sequences amplified from the 23 isolates and the sequence of GenBank accession NR112116 were aligned in Geneious v7.1.3. (Biomatters Ltd, New Zealand) using ClustalW platform (The alignment data can be accessed *via* DOI: 10.17632/cc6s8g4y2m.2.). The molecular phylogeny was reconstructed using the maximum likelihood method, as implemented in Randomized Axelerated Maximum Likelihood (Stamatakis et al.*,* 2014) adopting a General Time-Reversible model with branch support estimated using 1000 bootstrap (BS) pseudo replicates and rooted based on sister species *Bacillus subtilis*. The resulted phylogeny was visualized using FigTree v1.4.3. (Rambaut, 2016).

### 2.6 Determination of probiotic properties of the isolates

Fresh bacterial cultures grown using MRS agar at a 37 °C incubation temperature and 24 h of incubation time was used for probiotic property testing.

#### 2.6.1 Antibiotic sensitivity of the isolates

Antibiotic sensitivity was performed by disc diffusion method (Erginkaya et al., 2018) in duplicates using eight different antibiotics; gentamicin 30 μg/disc, erythromycin 15 μg/disc, amoxicillin 30 μg/disc, vancomycin 30 μg/disc, ampicillin 10 μg/disc, chloramphenicol 30 μg/disc, clindamycin 2 μg/disc and tetracycline 30 μg/disc (HiMedia). Two microliters of McFarland 0.5 standard solutions were prepared for the isolates and was spread on Muller Hinton Agar (MHA) plates (90 mm diameter) to conduct discs diffusion assay at an incubation temperature of 37 °C for 24 h.

#### 2.6.2 Determination of antimicrobial properties of the isolates

The antimicrobial properties of the isolates were determined in duplicates by agar well diffusion assay. First, McFarland 0.5 standard solutions were prepared using nine pathogenic species, i.e., *Candida albicans* (ATCC 90028), *Candida parapsilosis* (ATCC 22019), *Candida glabrata* (ATCC 90030), *Candida krusei* (ATCC 6258), *Candida tropicalis* (ATCC 13803), *Staphylococcus aureus* (NCTC 6571), *Escherichia coli* (NCTC 10418), *Salmonella enterica* serovar Typhi and *Shigella flexneri,* which were grown using MHA as a growth medium, incubated at 37 °C for 24 h.

From each prepared pathogen culture (McFarland 0.5 standard solutions), 2 mL were pipette out and flood inoculated onto MHA plates (90 mm diameter) and spread by rotating the plate. Then the wells were established on the MHA plate using a 9 mm sterile cork borer. Subsequently, the wells were loaded with 180 μL of cell-free supernatants of the isolates (pH adjusted for 4.5) prepared by centrifuging the MRS broth (consisted of 24 h grown bacteria) at 10,000 *×g* for 10 min. Finally, the plates were incubated at 37 °C for 24 h and the zone of inhibition around each well was measured. The inhibition diameter zones larger than 1 mm were considered as inhibitions resulting significant antimicrobial activity (Hilal Cadirci and Citak, 2005).

#### 2.6.3 Determination of bile tolerance of the isolates

The test was performed in duplicates, according to the method described by Song et al. (2015) using 1 mL of MRS broth containing fresh (24 h grown) bacterial cells per 9 mL of bile salt solution, a mean intestinal bile concentration of 0.3% (w/v) and a retention time of 4 h, identical to intestine conditions as suggested by Yavuzdurmaz (2007). Finally, the reduction of CFU (%) was calculated using the before and after incubation CFU counts.

#### 2.6.4 Stimulated gastric stress tolerance of the isolates

Resistance to pH 3 has been used during *in vitro* assays to study the resistance of the isolates to stomach pH. Moreover, food items stay generally 3 h in the stomach. Therefore, these conditions were taken into account during the experiment (Yavuzdurmaz, 2007). Tolerance to acid and pepsin (simulated gastric stress) was evaluated in duplicates using 9 mL of peptone water adjusted for pH 3, followed by adding pepsin adjusted to a final concentration of 1.5 g/L. One milliliter of cell suspensions consisted of fresh bacterial cultures (MRS broth contain bacterial cells) were added to the solution and allowed for incubation at 37 °C for 3 h. Finally, the reduction of CFU (%) was calculated using before and after incubation CFU counts.

### 2.7 Determination of milk coagulation ability of the isolates

Cell suspensions (1 mL) of the isolates were inoculated in duplicates into 10 mL of sterilized cow milk (as the control) and buffalo milk separately and incubated for 16 h at 42 °C (Hebert et al., 2001). The pH value of the sterilized milk before inoculation and after incubation was recorded. The milk coagulation ability of each isolate was observed using the pH of the milk in replicates at the end of the incubation period.

### 2.8 Statistical analysis

All the data were presented as mean ± SE from the duplicated analysis. ANOVA with Tukey post hoc test was performed using SAS (Statistical Analysis Software 9.0 version, SAS Institute Inc., Cary, North California, United States of America). The differences were considered significant at the probability of P < 0.05.

## 3 Results and discussion

### 3.1 Isolation and genus level identification of the isolates

**Figure 2:**
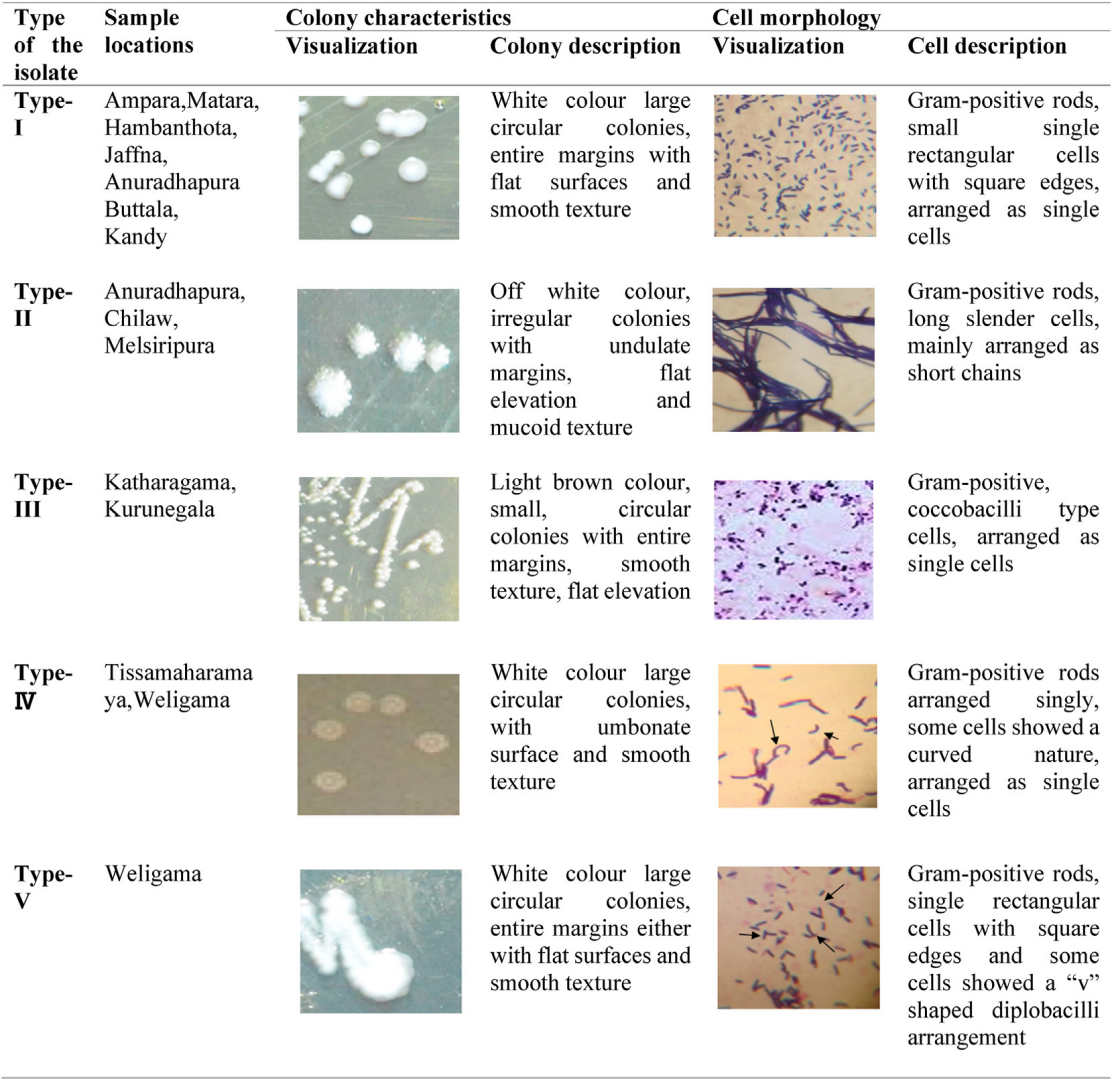
Colony characteristics and cell morphology of LAB isolated from Sri Lankan back-slopping *Meekiri.*

All isolates showed negative results for the catalase test, motility test, Voges –Proskauer test and H_2_S production. Moreover, the isolates were non-spore formers and showed fermentative sugar utilization patterns including glucose. The colony characteristics of the isolates differed in size, shape and appearance on MRS agar and five types of colonies were distinguished (Figure 2). Type-I, IV and V had white colour colonies, while type-II and III had off-white and light brown colonies. The smallest colony size (2–3 mm) was observed with type-III, while other isolates produced slightly larger size colonies (3–5 mm). The mucoid texture on the colony surface was observed with type-II, while others had a smooth texture. The majority of the isolates had flat surfaces, except for the type-IV, which had an umbonate colony surface. Except for coccobacilli shape in type-III, all other isolates had rod shaped cells. Type-II and V were observed with short chains and “v” shape diplobacilli cell arrangement, respectively, while other isolate types had a single-cell arrangement.

Based on the colony and cellular morphological differences, phenotypic heterogeneity (i.e. differences in the phenotype) of the LAB that lives as an isogenic microbial population in a homogenous environment (Sumner and Avery, 2002) was observed in the present study (Figure 2). Within a particular bacterial species, growth conditions and the growth stage of the cells may critically impact the cellular morphology. Similarly, phenotypic heterogeneity has been observed on MRS agar medium with species such as *L. fermentum*, especially under different stress conditions (Sung, 2005). Especially the genus *Lactobacillus* in LAB is highly heterogeneous as a result of high variation in the G + C content from 33 to 55% among its members (Belletti et al., 2009). It is generally considered that G + C content may vary among the members of a well-defined genus by not more than 10 % (Kant et al., 2011).

### 3.2 Molecular characterization and phylogenetic analysis of the isolates

**Figure 3:**
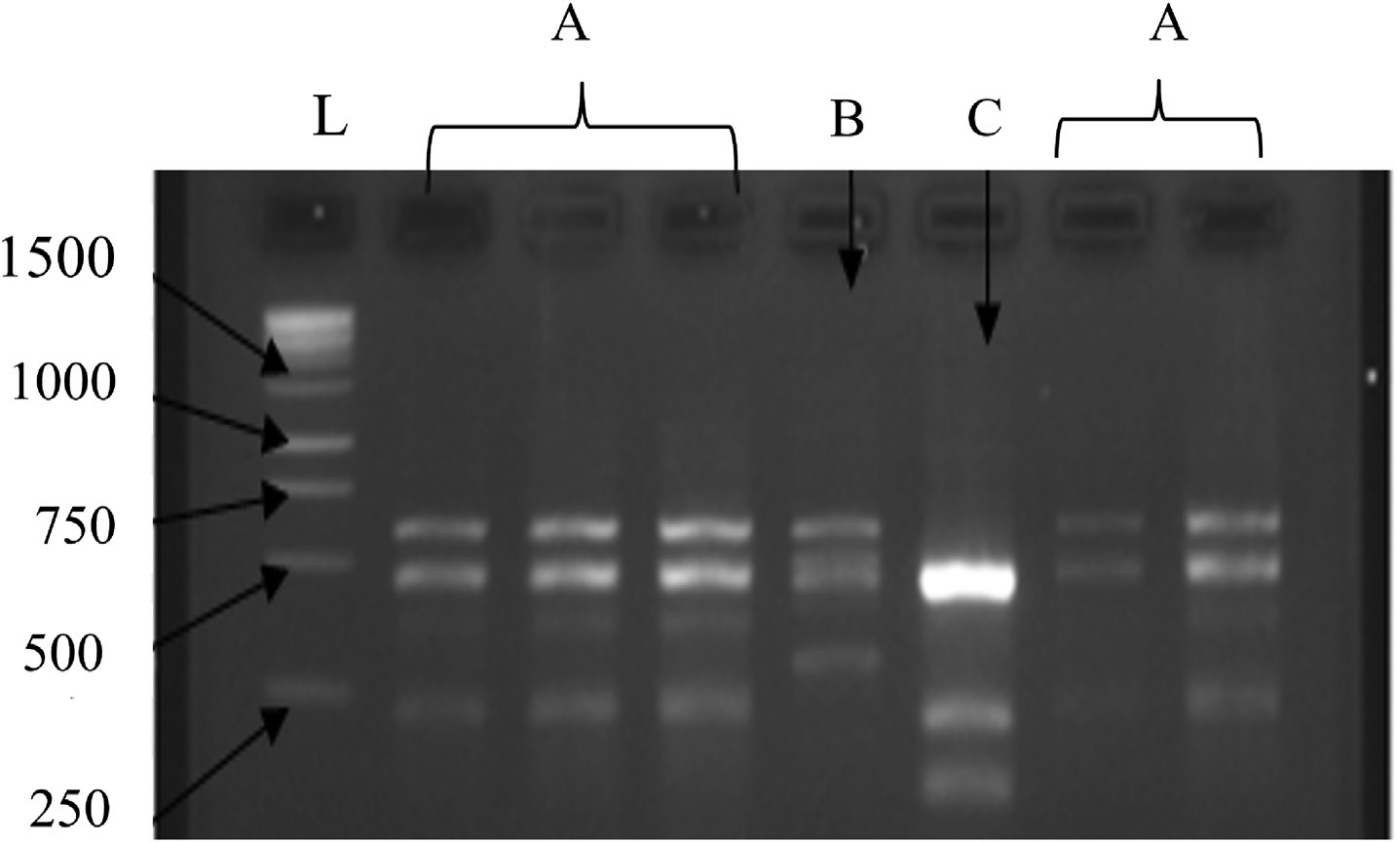
ARDRA patterns with *Hae*III restriction enzyme digestion of the LAB, isolated from back-slopping Sri Lankan *Meekiri*. A: *L. fermentum* and *L. plantarum*, B: *L. curvatus* and C: *L. acidophilus*, L = ladder (Promega, USA).

Traditionally, bacteria were identified using morphological and biochemical features (Hiremath et al., 2013). However, at present, exact identification is performed through molecular techniques because of the high precision and accuracy (Nataraja, 2016; Soto et al., 2010). Sequence homology and ARDRA are potent tools for grouping bacterial species into closely related groups. For ARDRA analysis the amplified 16S–23S rRNA intergenic spacer regions of isolated bacteria were digested with the restriction enzyme *Hae*III and finally, three prominent DNA banding patterns were observed (Figure 3).

Interestingly, according to the sequencing analysis results, all the isolates identified as *L. fermentum* and *L. plantarum* resulted in ‘A’ banding pattern in ARDRA, while the isolates *L. curvatus* and *L. acidophilus* resulted in ‘B’ and ‘C’ banding patterns, respectively. ARDRA is a technique, which aims at differentiating the microorganisms at species level, as it relies on the conserved nature of rRNA. However, it may not be sensitive enough to separate isolates accurately into species levels (Verlag et al., 2020). According to the observations in the present study, the ARDRA technique cannot be used to differentiate between *L. fermentum* and *L. plantarum*, however, can be used to differentiate *L. curvatus* and *L. acidophilus* from *L. fermentum* and *L. plantarum*. The use of several restriction enzymes together had shown better results in the identification processes. Therefore, it may be helpful for the identification of closely related species, as reported by Dimitonova et al. (2008) where a combination of *Sau*3A and *Hae*III restriction enzymes were used to separate *L. fermentum* and *L. plantarum* into distinguishable groups.

**Figure 4:**
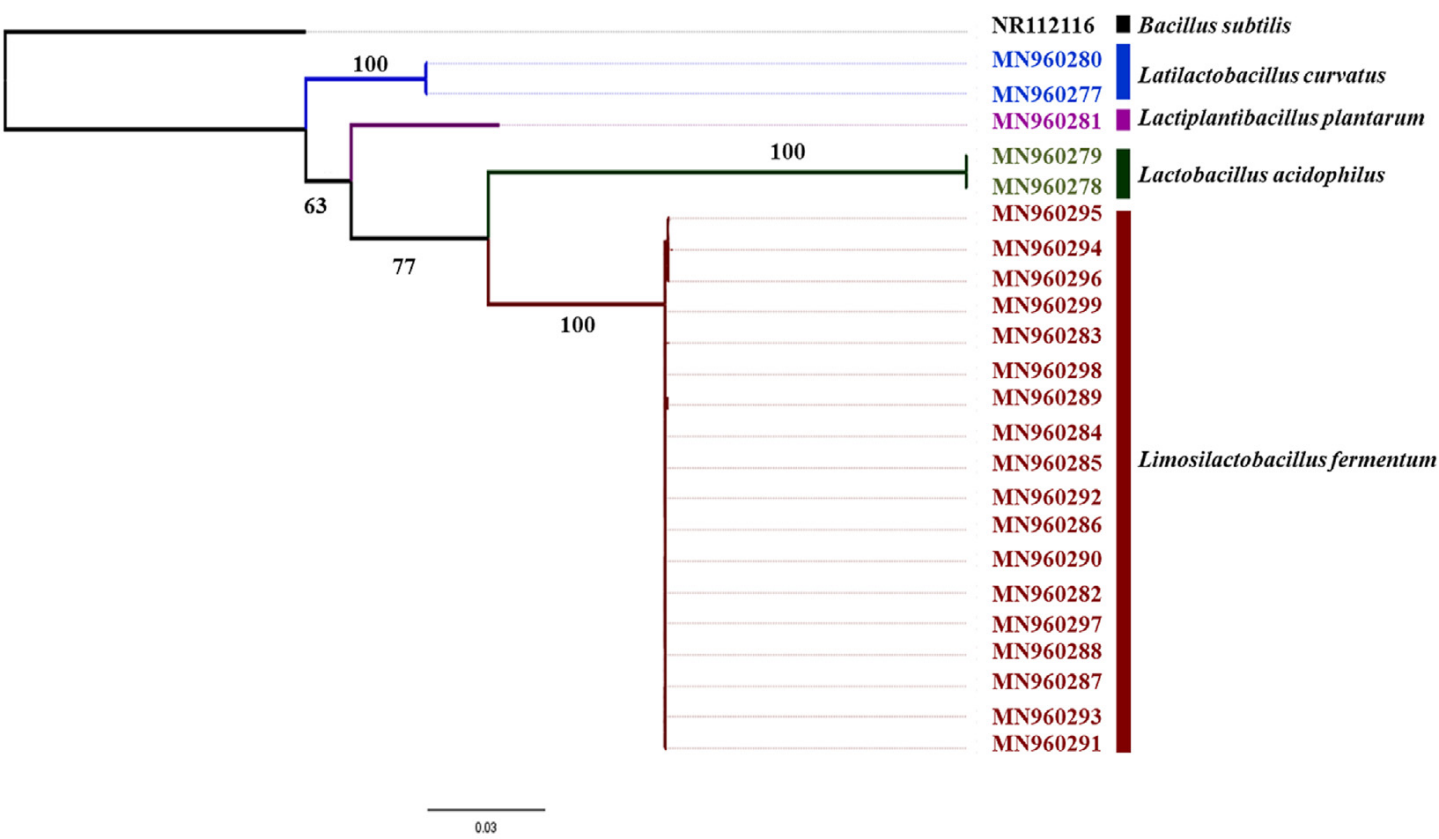
Phylogenetic tree describing the relationship of isolated LAB from Sri Lankan back-slopping *Meekiri*.

Finally, based on the sequence homology analysis of the 1500 bp PCR product, four species were identified namely; *Limosilactobacillus fermentum* (n = 18), *Latilactobacillus curvatus* (n = 2), *Lactobacillus acidophilus* (n = 2) and *Lactiplantibacillus plantarum* (n = 1). Earlier these species were named as *Lactobacillus fermentum*, *Lactobacillus curvatus*, *Lactobacillus acidophilus* and *Lactobacillus plantarum,* respectively. The validity of 16S rRNA gene sequencing on species identification depends on the length and quality of sequences. Generally, a minimum of 500–525 bp sequence that includes the more variable 5′-region is considered to be adequate for the identification of a group of bacteria (Drancourt et al., 2000), a criterion that was fulfilled in the present study. Furthermore, the phylogenetic tree reconstructed by rooting with the sister genus *Bacillus subtillis* as the out-group, assigned the isolates into four well-defined clades [bootstrap support >63], representing each of the four species identified in the study (Figure 4).

Based on sequence analysis, the common dairy starter species were not identified in the present study. This could be because the *Meekiri* samples were collected from traditional small-scale producers, who mainly used back-slopping to introduce LAB. Therefore, the origin of the isolated species could be raw milk (though a majority must be destroyed during the heat treatment at 90 °C for 10–15 min), milking utensils, or the processing environment (e.g. house flora), warranting the necessity to further investigate the origin of these species, enabling a full characterization of buffalo's milk value chain and isolation of LAB in every possible contamination step from milking to the production of *Meekiri*.

*L*. *plantarum* was isolated only from one sample collected from Jaffna, Sri Lanka (Table 1), while other species have no specificity to the sampling location. The fermented buffalo milk gel producers in the Jaffna area are engaged in fermented cow milk gel production as well. The same utensils are used for both operations, thus contaminations from cow milk may be a reason for the presence of *L. plantarum* only in the Jaffna sampling location. This is consistent with Vanniyasingam et al. (2019) where it was found that cow milk in the Jaffna area comprised of *L*. *plantarum* species*.*

**Table 1:** Physiological characteristics of LAB, isolated from back-slopping Sri Lankan *Meekiri.*

Species	Type of the species	Gene bank accession	Isolated geological region	Gas production from glucose	Sugar fermentation											Growth at different conditions						
																Temperature (˚C)				NaCl concentrations (%)		
					Arabinose	Galactose	Lactose	Maltose	Manitol	Melezitose	Melibiose	Raffinose	Salicin	Sorbitol	Trehalose	5	10	15	45	2	4	6.5
LF	I	MN960294	Ampara	+	-	+	+	+	-	-	+	+	-	-	+	-	-	+	+	+	+	+
		MN960282	Ampara	+	-	+	+	+	+	+	+	+	+	+	+	-	-	+	+	+	+	-
		MN960296	Hambanthota	+	-	+	+	+	-	-	+	+	-	-	+	-	-	+	+	+	+	-
		MN960299	Matara	+	-	+	+	+	-	-	+	+	-	-	+	-	-	+	+	+	+	+
		MN960289	Jaffna	-	-	+	+	+	-	-	+	+	-	-	+	-	-	+	+	+	+	+
		MN960285	Anuradhapura	+	-	+	+	+	+	+	+	+	+	+	+	-	-	+	+	+	+	+
		MN960286	Buttala	+	-	+	+	+	+	+	+	+	+	+	+	-	-	+	+	+	+	+
		MN960293	Kandy	+	-	+	+	+	-	-	+	+	-	-	+	-	-	+	+	+	+	+
		MN960291	Mathara	+	-	+	+	+	-	-	+	+	-	-	+	-	-	+	+	+	+	+
		MN960283	Mathara	+	-	+	+	+	-	-	+	+	-	-	+	-	-	+	+	+	+	+
		MN960291	Mathara	+	-	+	+	+	-	-	+	+	-	-	+	-	-	+	+	+	+	+
	II	MN960297	Anuradapura	-	-	+	+	+	-	-	-	-	+	-	+	-	-	-	+	-	-	-
		MN960284	Chilaw	-	-	+	+	+	-	-	-	-	+	-	+	-	-	-	+	-	-	-
		MN960295	Melsiripura	-	-	+	+	+	-	-	-	-	+	-	+	-	-	-	+	-	-	-
	III	MN960288	Katharagama	+	-	+	+	+	-	-	+	+	-	-	+	-	-	+	+	+	+	+
		MN960292	Katharagama	+	-	+	+	+	-	-	+	+	-	-	+	-	-	+	+	+	+	-
		MN960287	Kurunegala	+	-	+	+	+	-	-	+	+	-	-	+	-	-	+	+	+	+	-
		MN960298	Kurunegala	+	-	+	+	+	-	-	+	+	-	-	+	-	-	+	+	+	+	+
LA	IV	MN960278	Tissamaharamaya	-	-	+	+	+	-	-	-	-	+	-	-	-	-	-	+	+	+	-
		MN960279	Weligama	-	-	+	+	+	-	-	-	-	+	-	-	-	-	-	+	+	+	-
LC	V	MN960280	Weligama	+	-	+	+	+	-	-	-	-	+	-	-	-	-	+	+	+	+	+
		MN960277	Weligama	+	-	+	+	+	-	-	-	-	+	-	-	-	-	+	+	+	+	+
LP	I	MN960281	Jaffna	v	+	+	+	+	-	-	+	+	+	-	+	-	-	+	+	+	+	-

Limosilactobacillus fermentum (LF); *Lactobacillus acidophilus* (LA); Latilactobacillus curvatus (LC); Lactiplantibacillus plantarum (LP).

(+): positive reaction, (-): negative reaction, (V): variable, where isolates showed both positive and negative results when the experiment was conducted in duplicate.

*L. fermentum, L. plantarum* and *L. acidophilus* were isolated in most of the fermented buffalo milk products worldwide (Abesinghe et al., 2020; Harnentis et al., 2019; Pathak and Dutta, 2016). Moreover, *L*. *fermentum* is abundant in West Sumatran buffalo milk (Melia et al., 2018) which leads to abundance of *L. fermentum* in West Sumatran fermented buffalo milk, as reported in the current study.

### 3.3 Physiological characterization of the isolates

All the 23 isolates were subjected to physiological characterization and evaluation of probiotic properties. They showed variations in physiological characterization independent from sampling location and thus the same bacterial species from different sampling locations behave differently for the same test (Table 1).

#### 3.3.1 Gas production from glucose

According to the gas production from glucose, all the isolates were grouped into three groups (Table 1), homofermenters (no gas production from glucose); heterofermenters (positive gas production from glucose) and facultative heterofermenters (gas production from glucose was variable when repeating the test). Homofermentative species produce lactic acid as the sole product from carbohydrates, while heterofermentative species produce acetic acid, ethanol and CO_2_, in addition to lactic acid. Facultative heteroformentatives use glucose through Embden-Meyerhof pathway to produce lactic acid (Zhang and Vadlani, 2015). In this study, *L. fermentum* can be categorized as facultative heterofermentative species, as they gave positive and negative results depending on the isolate type and thus can act as either homofermenter or heterofermenter according to the situation. Furthermore, the gas production pattern of *L. fermentum* in the present study varied according to their type mentioned in Figure 2, even though all of them belongs to the same species. It is indicative of strain level or subspecies level differences as depending on the strain or subspecies level gas production pattern can be changed. Moreover, *L. curvatus* and *L. acidophilus* were identified as a heterofermenter and a homofermenter, respectively, while *L. plantarum* was not classified to any of the above-mentioned groups due to its variable behavior in gas production.

#### 3.3.2 Carbohydrate utilization profiles of the isolates

The type of carbohydrate and the species of microorganism used during the fermentation is an important feature in fermented product processing, as it contributes to the development of aroma, flavour and the preservation of the final product. Rapid production of acids at the expense of carbohydrates is a key characteristic expected from a potential starter culture. In the present study, all the isolates are capable of utilizing galactose, lactose and maltose. Hence, all of them have the potential to ferment milk, as lactose is the main fermentable sugar present in milk. In the current study, isolates performed differently in fermenting sugars. These variations among the species may be due to their plasmid-encoded character, which in such situations result loss or gain of plasmids. This can lead to inconsistencies in metabolic characters resulting different fermentation results (Belletti et al., 2009).

#### 3.3.3 Temperature tolerance of the isolates

According to the growth at different temperatures, none of the isolates was able to grow at 5 °C and 10 °C, while all grew at 45 °C. At 15 °C the growth of the isolates varied (Table 1). The thermophilic bacteria have their optimum growth at 37 °C–45 °C and are mainly used in the production of yoghurt, fermented buffalo milk, acidophilus milk and Swiss-type cheese (De Angelis et al., 2004). Since Sri Lanka is a tropical country and *Meekiri* are stored at room temperature (approx. 35 °C), all the isolates observed in the present study can grow at temperatures around 35 °C. Lactic acid bacteria, especially *Lactobacillus* species have special mechanisms for heat resistance which involves enhancing the activity of chaperones; the highly conserved stress proteins that confer enhanced resistance to elevated temperatures, ribosome stability, temperature sensing, and control of ribosomal function at high temperatures (De Angelis et al., 2004).

#### 3.3.4 NaCl tolerance of the isolates

Type-II, *L. fermentum* species were not able to tolerate NaCl concentrations, while the majority of the other *L. fermentum* isolates could tolerate NaCl concentrations up to 6.5% (Table 1). Similarly, the *L. fermentum* species isolated from buffalo milk in Bangaladesh showed tolerance to NaCl concentration from 1-6% and no growth from 8-10% NaCl concentrations (Forhad et al., 2015). Therefore, within the species the ability to tolerate NaCl can differ. Similarly, Reque et al. (2000) confirmed that *L. fermentum* can grow at a salt concentration of up to 7%. In the current study, *L. curvatus* could tolerate NaCl concentration up to 6.5% and similarly, Verluyten et al. (2004) identified that *L. curvatus* can grow at 7% of NaCl concentrations. However, the activities such as bacteriocin production reduced at that level. The ability of LAB to tolerate NaCl concentrations at least up to 2% can be useful as some dairy products (butter) require exposure to salt concentrations (Khedid et al., 2009). However, in the current study, the NaCl tolerance of the isolates varied depending on the isolated geological region and this needs to be addressed in future studies.

### 3.4 Determination of potential for probiotic properties of the isolates

#### 3.4.1 Antibiotic sensitivity of the isolates

We found all the isolated *L. fermentum* and *L. plantarum* were sensitive to the studied antibiotics, except vancomycin, while *L. acidophilus* and *L. curvatus* isolates were sensitive to all the studied antibiotics (Table 2). Bacterial strains intended to be used as probiotics during food production must be examined for antibiotic susceptibility to avoid the spread of antibiotic resistance via the food chain. Therefore, antibiotic resistance is considered as a negative characteristic in selecting novel bacteria species as potential probiotics (Ashraf and Shah, 2011). The detection of many antibiotic resistance genes assorted with human gut microbiome suggests that antibiotic-resistant bacteria in the gastrointestinal tract function as an antibiotic resistance gene pool (Fukao et al., 2009). Vancomycin resistance is not considered negatively for potential probiotics, as it is a non-transferrable, intrinsic type of resistance often associated with many other species (Ahmadova et al., 2013).

**Table 2:** Probiotic properties of LAB, isolated from Sri Lankan back-slopping *Meekiri.*

Species and the type	Antibiotic sensitivity								Antimicrobial property (average inhibitory zone in mm)						Bile acid tolerance (%reduction of CFU)	Acid tolerance (% Reduction of CFU)
	Gentamicin	Erthromycin	Amoxicillin	Vancomycin	Ampicillin	Chloramphenicol	Clindamycin	Tetracycline	*Salmonella enterica* serovar Typhi	*Shigella flexneri*	*E.coli* NCTC 10418	*Staphylococcus aureus* NCTC 6571	*Candida krusei* (A TCC 6258)	*Candida tropicalis* (ATCC 13803)		
LF (I)	S	S	S	R	S	S	S	S	04.75 ± 1.1^b^	09.75 ± 0.4^a^	02.00 ± 0.7^a^	05.25 ± 0.3^a^	09.00 ± 1.4^a^	05.50 ± 0.7^a^	76.92	11.92
LF (II)	S	S	S	R	S	S	S	S	05.75 ± 1.1^b^	08.75 ± 0.4^a^	01.75 ± 0.4^a^	06.25 ± 0.3^a^	05.25 ± 1.1^a^	05.00 ± 1.4^a^	64.11	09.05
LF (III)	S	S	S	R	S	S	S	S	07.00 ± 1.4^b^	07.75 ± 1.1^a^	02.25 ± 0.4^a^	05.00 ± 1.4^a^	06.75 ± 1.1^a^	05.75 ± 1.1^a^	70.71	09.89
LA (IV)	S	S	S	S	S	S	S	S	12.75 ± 0.3^a^	13.25 ± 1.1^a^	02.75 ± 0.4^a^	10.25 ± 1.1^a^	10.75 ± 1.1^a^	04.75 ± 0.3^a^	07.34	13.99
LC (V)	S	S	S	S	S	S	S	S	11.00 ± 0.7^a^	13.75 ± 1.8^a^	02.50 ± 0.7^a^	09.25 ± 1.8^a^	07.75 ± 2.5^a^	06.25 ± 1.1^a^	55.05	29.47
LP (I)	S	S	S	R	S	S	S	S	10.75 + 1.1^a^	14.25 + 1.8^a^	02.75 + 1.1^a^	10.5 + 2.8^a^	07.25 + 1.8^a^	05.75 + 1.1^a^	07.47	38.92

Results are shown as mean ± SE. Means within the same column bearing different superscripts are significantly different (p < 0.05). CFU: colony-forming units *Limosilactobacillus fermentum* (LF); *Lactobacillus acidophilus* (LA); *Latilactobacillus curvatus* (LC); *Lactiplantibacillus plantarum* (LP); S = sensitive, R = resistance.

#### 3.4.2 Antimicrobial activity of the isolates

All the isolates demonstrated antimicrobial activity against *Salmonella enterica* serovar Typhi*, Shigella flexneri, E. coli* (NCTC 10418) and *Staphylococcus aureus* (NCTC 6571)*.* Furthermore, *L*. *plantarum* isolated from *Dadih* in West Sumatra was identified with good probiotic properties given it inhibits the growth of *E. coli* (Abesinghe et al., 2020); however, such growth inhibition of *E. coli* was not observed in the current study and it may be a strain level differences within the same species*. L. acidophilus* and *L. curvatus* showed the highest antimicrobial ability against *Salmonella enterica* serovar Typhi (Table 2) in agreement with Makras et al. (2006). Moreover, the isolates demonstrated antifungal activity against *Candida krusei* (ATCC 6258) and *Candida tropicalis* (ATCC 13803). The preserving effects rendered are mainly due to the production of lactic acid, acetic acid and other compounds such as ethanol, formic acid, hydrogen peroxide, diacetyl, reuterin, reutericyclin and bacteriocin or related compounds (Anas et al., 2010). In contrast, Rönnqvist et al. (2007) found that *L. fermentum* (strain Ess-1) produce antifungal agents against *C. albicans;* however, the antifungal effect for *C*. *albicans* was not evident in the current study with respect to any of the *L. fermentum* strains. Therefore, it is possible to conclude in some antimicrobial and antifungal properties differ at strain level.

#### 3.4.3 Bile tolerance of the isolates

*L. acidophilus* together with *L. plantarum* showed higher bile acid tolerance compared to others (Table 2). Similarly, Song et al. (2015) found no CFU reduction in *L. acidophilus* under 0.3% bile acid. However, *L. fermentum* and *L. acidophilus* strains isolated from *Dadih* in Western Sumatra showed an acceptable level of 0.3% bile acid tolerance (Melia et al., 2018). Growth retardation of bacteria in bile acid is acceptable, as cellular homeostasis is disrupted by bile salts and thus leads to the dissociation of the lipid bilayer and integral proteins of cell membranes. It results in the leakage of bacterial cell content and finally cell death (Begley et al., 2005). Bile acid tolerance of the isolates depends on their colony and cell morphology (Pan et al., 2009) and it can be the reason for various CFU% reductions in the current isolates though phenotypic heterogeneity associated with bile tolerance was not evaluated in the present study.

#### 3.4.4 Stimulated gastric stress tolerance of the isolates

Probiotics have to be survived in the gastric acid environment to colonize the small intestine, hence, acid tolerance is considered as a desirable property when selecting a potential probiotic strain (Corcoran et al., 2005). In the present study, acid tolerance is comparatively low in *L. curvatus* (Table 2) and high in *L. fermentum*. Apart from that *L. fermentum* isolated from *Dadih* showed a 57.1% of survival rate under pH 4 and it's lesser at pH 3 (Amelia et al., 2020). Changes in cellular morphology and cell surface hydrophobicity have been observed with acid and osmotic stress in *L. fermentum* (Sung, 2005), and it may be the reason for the high acid tolerance of *L. fermentum* reported in this study*.* Lactic acid bacteria such as *Lactobacillus* spp. are intrinsically resistant to acid, however, they exhibit an increased sensitivity at pH levels below three.

### 3.5 Determination of milk coagulation ability of the isolates

In the current study, the ability of the isolates to coagulate cow's milk (control) and buffalo's milk while reducing the milk pH was evaluated (Table 3). *L. fermentum* (Type-III), *L. curvatus* and *L. acidophilus* showed the highest (p < 0.05) pH reduction in both cow's and buffalo's milk. In *L. curvatus* species several genes associated with metabolic pathways encoding proteins that are involved with carbohydrate utilization were identified (Chen et al., 2020) and it may result in high production of acids in milk leading to a high pH reduction as seen in the present study. In both situations, *L. fermentum* (Type-I), showed the lowest (p < 0.05) pH reduction and it may be associated with its facultative heterofermentative nature which uses substrates to produce both acid and non-acidic substances depending on the situation. The ability to coagulate milk within 16 h at 42 °C with 1% fresh inoculum is considered a useful characteristic in bacteria expected to be used for the production of various fermented dairy products (fast milk coagulators) (Hebert et al., 2001). Casein, the structural milk protein is organized as micelles and it is stable in its natural form of milk. However, changing the natural milk conditions (e.g. decrease the pH of milk) neutralize the charges of casein micelles, destabilize and precipitate the caseins once it reaches the isoelectric point (approximately pH 4.6), and thus milk coagulation occurs (Sinaga et al., 2017). None of the isolated species in the study was identified as fast milk coagulators as none of them coagulates milk completely at 42 °C within 16 h.

**Table 3:** pH variation of raw milk (buffalo's milk and cow's milk as the control) by isolated LAB from Sri Lankan back-slopping *Meekiri.*

Species and the type	pH of Cow milk		pH of Buffalo milk		pH reduction in cow milk	pH reduction in buffalo milk
	Before incubation	After incubation	Before incubation	After incubation		
LF (I)	6.42 + 0.01	5.41 + 0.02	6.64 + 0.02	5.62 + 0.04	1.01 ± 0.03^d^	1.02 ± 0.02^d^
LF (II)	6.45 + 0.01	5.17 + 0.01	6.57 + 0.01	5.26 + 0.04	1.28 ± 0.02^c^	1.31 ± 0.03^c^
LF (III)	6.43 + 0.03	4.84 + 0.00	6.60 + 0.00	5.12 + 0.11	1.59 ± 0.03^a^	1.48 ± 0.11^a^
LA (IV)	6.42 + 0.06	4.89 + 0.02	6.53 + 0.02	5.06 + 0.03	1.53 ± 0.04^a^	1.47 ± 0.05^a^
LC (V)	6.48 + 0.08	4.86 + 0.00	6.58 + 0.00	5.06 + 0.11	1.62 ± 0.08^a^	1.52 ± 0.11^a^
LP	6.45 + 0.00	5.02 + 0.02	6.53 + 0.02	5.15 + 0.03	1.43 + 0.02^b^	1.38 + 0.01^b^

Results are shown as mean ± SE. The incubation period was 16 h.

Means within the same column bearing different superscripts are significantly different.

(p < 0.05).

## 4 Conclusions and future perspectives

Sri Lankan back-slopping *Meekiri* is an excellent source of LAB such as *L. fermentum, L. curvatus, L. acidophilus* and *L. plantarum.* Isolates could vigorously grow at 45 °C and tolerate NaCl up to 6% (w/v). Lactic acid bacteria species found in the studied back-slopping *Meekiri* were distributed across Sri Lanka without local specificity; however, their properties and characteristics varied with sampling locations. Identified isolates demonstrated promising probiotic potential with great antibiotic sensitivity and antimicrobial activity. Moreover, the isolated species possessed optimal acid and bile tolerance *in vitro*. The current study cast light on potential LAB species commonly found in back-slopping *Meekiri* and future research should focus on high throughput sequencing technologies to identify their detailed taxonomic categories*.* Continuous use of back-slopping results in the selection of best-adapted strains under the given conditions and those strains can be improved as starter cultures. Therefore, further studies are recommended with a larger sample size, emphasizing proximate composition, pH and organoleptic properties of back-slopping *Meekiri* for a thorough evaluation of the microbial diversity to be used as starter cultures.

## Declarations

### Author contribution statement

A. M. M. U. Adikari: Conceived and designed the experiments; Performed the experiments; Analyzed and interpreted the data; Wrote the paper.

Hasitha Priyashantha, D. V. Jayatileka, S. P. Kodithuwakku, J. A. M. S. Jayatilake, J. K. Vidanarachchi: Conceived and designed the experiments; Analyzed and interpreted the data; Contributed reagents, materials, analysis tools or data; Wrote the paper.

J. N. K. Disanayaka: Conceived and designed the experiments; Analyzed and interpreted the data; Wrote the paper.

### Funding statement

This work was supported by 10.13039/501100011612University Grants Commission – Sri Lanka and 10.13039/501100001724International Foundation for Science (B-5367-1).

### Data availability statement

Data included in article/supplementary material/referenced in article.

### Declaration of interests statement

The authors declare no conflict of interest.

### Additional information

No additional information is available for this paper.
